# Knowledge and Opinions of Secondary School Teachers on Teaching the Subject of Sex Education

**DOI:** 10.7759/cureus.98828

**Published:** 2025-12-09

**Authors:** Kyriaki Evdaimon, Kleanthi Gourounti, Athina Diamanti, Victoria Vivilaki, Angeliki Sarella

**Affiliations:** 1 Department of Midwifery, University of West Attica, Athens, GRC; 2 Department of Health and Welfare, 1st Vocational High School of Koropi, Attica, GRC

**Keywords:** educational seminars, secondary school teachers, sex education, sexual education, sexual health

## Abstract

Purpose: The study aims to investigate the knowledge and opinions of secondary school teachers regarding the sexual and reproductive health of adolescents and the teaching of the sex education course.

Methods: The research was carried out with a structured questionnaire. Samples were first pilot tested, and the value of the Cronbach alpha index was calculated, which highlights its reliability since it was equal to 0.72. The statistical analysis was carried out using IBM SPSS Statistics for Windows, Version 26.0 (Released 2019; IBM Corp., Armonk, New York, United States), where, for the questions with a Likert-type scale, the frequency, percentage, median, and interquartile range were calculated, while, for the categorical variables, the frequency and percentage were calculated. To investigate whether a quantitative variable differed between two levels, a t-test was used, and to investigate correlations between quantitative variables, the value of the Pearson linear correlation coefficient was calculated. The statistical significance level for all tests was 5%.

Results: The study included 192 secondary school teachers with an average age of 48.04 years, and 124 (64.4%) were women. The average time in service was 15.14 years, and 39.6% (n=76) worked in schools in the Attica region. One hundred and sixty (83.2%) need additional training and want both appropriate educational material (93.6%) and training seminars (90.8%). One hundred and sixty-five (85.7%) believe that sex education should be taught by qualified teachers, and 39.3% (n=75) of teachers consider that high school is the most appropriate level of education, while 37.7% (n=72) consider primary school. The majority of the sample believes that sex education should be taught as a subject integrated into the curriculum of other subjects (65.7%), as a chapter of health education (63.5%), or as an independent subject (57.8%). Comparatively, the level of knowledge between teachers in the health sector and those in other specialties, having taken into account the 156 teachers who had answered the specific questions, appears to differ (t(156)=-3.61; p<0.001), with the former having a higher level of knowledge. Finally, the level of knowledge in the psychosexual development of children differs between teachers who have attended a training program related to sex education and those who have not (t(156)=- 2.35; p=0.020), with the former again having a higher level of knowledge.

Conclusion: The study highlights that, while educators recognize the importance of sex education (ideally delivered by qualified staff), there is a significant lack of adequate training and educational resources. The finding that teachers with a health background or previous training demonstrated a statistically higher knowledge level (p<0.001 and p=0.020, respectively) emphasizes the critical need for organized seminars and certified materials to ensure the effective implementation of the curriculum in schools.

## Introduction

Health is one of the fundamental rights. The World Health Organization (WHO) defines it as "a state of complete physical, mental, and social well-being and not merely the absence of disease or infirmity" [1]. In this context, prevention emerges as a cornerstone for the protection and promotion of health. Primary prevention, focusing on information and education, shields individuals and groups against diseases and harmful behaviors [2]. Health education programs, by strengthening knowledge, skills, and healthy attitudes, are a key tool for prevention [3]. The adoption of healthy habits and a responsible attitude towards health ensure individual and social well-being, laying the foundations for a healthy and productive future [4].

Health education, based on scientific foundations, empowers individuals and societies to adopt healthy behaviors [5]. The school is the ideal place for its implementation, because it shapes positive attitudes and knowledge from a young age [6].

In this context, sex education programs in schools play a primary role as timely and valid information that shields adolescents against risky sexual behaviors, promoting their health and well-being. Several studies have demonstrated high rates of teenage pregnancy. It is also important to emphasize the frequent occurrence of sexually transmitted diseases, which demonstrates the urgent need to improve sex education in schools [7,8]. This study aims to assess the knowledge, attitudes, and training needs of Greek secondary school teachers regarding sexual and reproductive health education and to identify the factors that influence teachers' preparation for teaching this subject.

This article was previously presented as a meeting abstract at the 26th Congress of the World Association for Sexual Health on November 2-5, 2023.

## Materials and methods

This research was conducted online, with an anonymous, structured questionnaire (see Appendices; only the questions related to the specific topic are included). An approval from the director of each school was obtained before submitting the questionnaire to the teachers. On the website, there was a formal informed consent as well as a research participation form in order to ensure the informed consent of all participating teachers. Also, all the necessary information for completing the questionnaire was provided, including the purpose and necessity of the research and assurances regarding the anonymity of the participants. Those participants who wanted to express complaints about this study had the opportunity to do so, either by sending an email to the address indicated on the consent and participation form in the research or by completing the corresponding complaint form.

The study involved secondary school teachers (middle school, high school, vocational high school) who have been assigned to teach health education courses (biology, health education) (inclusion criteria) and work in schools in Greece (both rural and urban areas). All the other teachers not teaching these courses were excluded.

The questionnaire consists of four parts that record the following: (a) sociodemographic data, (b) knowledge on sexual and reproductive health issues, (c) results of health education programs that have been implemented to date among adolescents, and (d) views on the necessity of organizing training programs on sexual and reproductive health issues for secondary education teachers. The creation of the questionnaire was based on a literature review and was initially tested on individuals in order to ensure its validity and reliability. At the same time, the value of the Cronbach alpha index was calculated, which was equal to 0.72, in order to highlight the reliability of the research tool that was constructed for the needs of the research.

The statistical analysis of the resulting data was carried out using IBM SPSS Statistics for Windows, Version 26.0 (Released 2019; IBM Corp., Armonk, New York, United States). Descriptive and inferential statistics were used. For Likert-type scale questions, the frequency and percentage for each category, the median, and the interquartile range were calculated. For categorical variables, the frequency and percentage for each category were calculated. It is noted that to assess the level of teachers' knowledge regarding sexual and reproductive health, their total score on the questions asked was calculated, based on the correct/incorrect answers they gave. Finally, the total score on these questions was calculated by summing the individual values ​​of the variable. More specifically, to investigate whether a quantitative variable differed between two levels, a t-test for independent samples was used. In addition, to investigate correlations between quantitative variables that had a normal distribution, the value of the Pearson linear correlation coefficient was calculated. The statistical significance level for all tests was 5%.

## Results

The survey included 192 secondary school teachers with an average age of 48.04 years, and 124 (64.4%) were women. The average time in service was 15.14 years, and 39.6% (n=76) worked in schools in the Attica region. One hundred and sixty (83.2%) need additional training and want both appropriate educational material (93.6%) and training seminars (90.8%). One hundred and sixty-five (85.7%) believe that sex education should be taught by qualified teachers, and 39.3% (n=75) of teachers consider that high school is the most appropriate level of education, while 37.7% (n=72) consider primary school. Finally, the majority of the sample believes that sex education should be taught as a subject integrated into the curriculum of other subjects (65.7%), as a chapter of health education (63.5%), or as an independent subject (57.8%). 

Of the total 192 teachers, 62 work in the health sector, and 130 have other specialties. Taking into account the 156 teachers who had answered the specific questions in Table 1 and comparing them, we observe that the level of knowledge on sexual and reproductive health issues differs between teachers in the health sector and those in other specialties (t(156)=-3.61; p<0.001), with the former having a higher level of knowledge on the subject in question. Also, the level of knowledge on issues of medicine and gender biology (t(156)=-4.92; p<0.001), information on issues concerning the sexual behavior of modern people (t(156)=-2.00; p=0.047), and the ability to provide advice to students on issues of reproductive system anatomy, sexually transmitted diseases, and contraception (t(156)=-4.98; p<0.001) differ depending on the specialty of the teachers. More specifically, it is found that health sector teachers consider that they have a higher level of knowledge in matters of medicine and gender biology, can provide information to a higher degree on issues concerning the sexual behavior of modern people, and have a higher degree of ability to provide advice to students on issues of reproductive system anatomy, sexually transmitted diseases, and contraception, compared to teachers of other specialties. 

**Table 1:** T-test results for investigating differences in the level of knowledge of teachers on sexual and reproductive health issues depending on whether or not they attended a relevant training program (p<0.05 and p<0.001) The numbers in bold highlight statistically significant research results. SD: standard deviation; AP: mean value

Teachers' knowledge of sexual and reproductive health issues	Attending a training program on sexual and reproductive education				t(156)	p
	No		Yes			
	Mean	SD	AP	SD		
Level of knowledge on sexual and reproductive health issues	6.23	3.37	7.19	3.91	-1.66	0.099
Level of knowledge in medical and gender biology issues	3.14	0.97	3.27	0.95	-0.81	0.418
Level of knowledge in the psychosexual development of children	2.65	0.85	2.97	0.85	-2.35	0.020
Information on issues concerning the sexual behavior of modern people	2.97	0.90	3.31	0.87	-2.42	0.017
Ability to handle new technologies, such as the internet, to obtain information/material	4.14	0.85	4.25	0.77	-0.84	0.400
Training in the use of new teaching approaches	3.58	1.03	3.90	0.82	-2.15	0.033
Personal comfort for discussing sexual and reproductive issues in the classroom	3.63	1.20	3.99	1.08	-1.97	0.051
Ability to advise students on issues of reproductive system anatomy, sexually transmitted diseases, and contraception	3.42	1.01	3.96	0.84	-3.64	<0.001

Furthermore, taking into account the 156 teachers who had answered the specific questions in Table 2 and comparing them, it emerges that the level of knowledge in children's psychosexual development differs between the 42.4% of teachers who have attended a training program related to sexual and reproductive education and the 57.6% of teachers who have not attended (t(156)=-2.35; p=0.020), with the former having a higher level of knowledge in children's psychosexual development. In addition, teachers' opinions regarding information on issues related to the sexual behavior of modern people (t(156)=-2.42; p=0.017) and training in the use of new teaching approaches (t(156)=-2.15; p=0.033) differ depending on whether or not they have attended a relevant training program. In particular, it appears that teachers who have attended a training program related to sexual and reproductive education consider that they have a higher level of knowledge in the psychosexual development of children, can provide information to a greater extent on issues related to the sexual behavior of modern people, and have greater training in the use of new teaching approaches, compared to teachers who have not attended. 

**Table 2:** T-test results for investigating differences in the level of knowledge of teachers on sexual and reproductive health issues depending on their specialty (p<0.05 and p<0.001) The numbers in bold highlight statistically significant research results. SD: standard deviation; AP: mean value

Teachers' knowledge of sexual and reproductive health issues	Teachers of other specialties		Health educators		t(156)	p
	Mean	SD	AP	SD		
Level of knowledge on sexual and reproductive health issues	5.85	3.51	7.71	3.44	-3.61	<0.001
Level of knowledge in medical and gender biology issues	2.91	0.90	3.56	0.87	-4.92	<0.001
Level of knowledge in the psychosexual development of children	2.69	0.86	2.91	0.81	-1.73	0.085
Information on issues concerning the sexual behavior of modern people	2.97	0.88	3.24	0.93	-2.00	0.047
Ability to handle new technologies, such as the internet, to obtain information/material	4.16	0.82	4.01	0.94	1.16	0.247
Training in the use of new teaching approaches	3.75	0.96	3.53	0.97	1.56	0.121
Personal comfort for discussing sexual and reproductive issues in the classroom	3.62	1.20	3.87	1.03	-1.49	0.137
Ability to provide advice to students on reproductive system anatomy, sexually transmitted diseases, and contraception	3.40	0.97	4.05	0.82	-4.98	<0.001

## Discussion

Teachers who had training reported higher knowledge of sexual and reproductive health. According to this study and the recent similar study by Ladopoulou carried out in 2022, gaps in their knowledge emerged, capable of creating problems in the validity of the information they provide to students. These gaps seem particularly greater when it comes to teachers of other specialties beyond the health sector, as well as for teachers who have not been trained by attending a relevant seminar [9,10]. This, therefore, highlights the need to improve knowledge and impose better preparation of teachers, so that they become competent in teaching sex education and, ultimately, in the adoption of healthy strategies by adolescents [11,12].

Improving educational competencies enhances students' learning and, consequently, social outcomes, by acquiring the knowledge and skills required to adapt, practice, and maintain healthy behaviors throughout their lives [13,14]. Teachers report that they are forced to address concerns and behaviors of sexual content among their students and thus have to include sex education in their teaching work and recognize, in their majority, the necessity of organizing training programs in order to ensure effectiveness in their educational task [15,16]. Furthermore, according to the views of the teachers expressed in this study, a very large percentage of them believe that sex education should be taught by teachers trained in sexual and reproductive health issues, through attending appropriate training seminars. In fact, they consider the most appropriate level of education to be the high school firstly and then the primary school [17].

Furthermore, it is evident that most of the teachers support that the sex education course should be included in the curriculum of other subjects. With very small differences, of course, they seem to support that it should be taught as a chapter of a new subject, health education, followed by the view that it should constitute a unique, separate subject [18]. At the same time, the barriers faced by sex education teachers need to be overcome, such as the limitations and guidance of the curricula, the concerns and reactions of parents, the school administration, and the religious beliefs, in order to enable teachers to contribute significantly to the lives and well-being of students [19].

In the sample of teachers examined, an attempt was made to include teachers from both urban and rural areas. The limitations of the study concern the sample size and that the answers to some of the questions were based on the subjective assessments of the respondents themselves.

## Conclusions

The findings of this research reinforce the importance of the existence of sex education in educational work. However, teachers, as the primary mediators of knowledge, recognize the central position of sex education in the educational process and emphasize the need for continuous training so that they can adequately respond to the demands of this extremely important field. Collaboration with health professionals, such as doctors, midwives, and other health professionals within the framework of interdisciplinarity, could benefit both in the conduct of training seminars for teachers by them and in the co-teaching of the sex education course.

Today's Greek school needs to be ready and mature to welcome this course. Teachers show a positive attitude as they fully understand the necessity of providing comprehensive sex education to students. They recognize that sex education is a critical element for the holistic development of young people, contributing to the cultivation of healthy relationships, risk prevention, and the empowerment of individuals. In addition, students demonstrate their interest through the expression of sexual questions and behaviors; through their active participation in discussions regarding sexual issues; by seeking information in various ways, such as the internet; by expressing concerns and fears about sexuality, relationships, or body image; and by showing interest in sexual partners and experimentation.

The benefits of including sex education in schools will be varied; therefore, communication between teachers and parents should be improved, teacher training should be provided, and curricula should be redefined to include this subject.

## Appendices

**Table 3:** Knowledge of sexual and reproductive health

	Not at all	Little	Moderate	Enough	Absolutely well
To what extent do you think you are aware of sexual and reproductive health?					

**Table 4:** Note the region where the school unit you belong to/work is located

Crete		Attica		Thessaloniki	
Macedonia		Thrace		Epirus	
Central Greece/Evia		Thessaly		Peloponnese	
Ionian Islands		Aegean Islands		Other:	

**Table 5:** Level of education/basic degree

Basic degree			
Medicine	Nursing/obstetrics	Aesthetics	Medical laboratories
Dental technology	Social work	Radiology	Physiotherapy
Nursery	Public hygiene	Physics	Chemistry
Physiognomy	Biology	Geology	Agriculture
Level of education			
	Higher education	Master's degree	PhD

**Table 6:** Level of knowledge of various subjects

In your opinion, how is your level of knowledge in…					
	Not good at all	Not so good	Good	Very good	Extremely good
Medical and gender biology issues?					
The psychosexual development of children?					
Information on issues related to the sexual behavior of modern people?					
The ability to manipulate new technologies, such as the internet, to obtain information/material?					
Training to use new teaching approaches?					
The personal comfort of discussing sexual and reproductive issues in class?					

**Table 7:** Advisory to students

	Not at all	Little	Moderate	Enough	Absolutely well
To what extent do you think you could advise your students on issues of reproductive system anatomy, sexually transmitted diseases, and contraception?					

**Table 8:** Reeducation need

	Yes	No
Have you attended any training program related to sexual and reproductive education?		
